
JOURNAL INFORMATION
==============================
NLM Title Abbreviation: ScientificWorldJournal
Journal ID: ScientificWorldJournal
NLM Journal ID: 101131163
Journal ID: TSWJ

The Scientific World Journal

ISSN: 2356-6140
EISSN: 1537-744X
Publisher: Wiley

ARTICLE INFORMATION
==============================
PMCID: PMC5823126
PMID: 19219377
DOI: 10.1100/tsw.2009.12
Article ID: 640506
Article version: 1
Subjects: Directions in Science

Mature miR-184 and Squamous Cell Carcinoma of the Tongue

Wong Thian-Sze *
Ho Wai-Kuen
Chan Jimmy Yu-Wai
Ng Raymond Wai-Man
Wei William Ignace
Department of Surgery, Faculty of Medicine, University of Hong Kong, China
*Thian-Sze Wong: thiansze@graduate.hku.hkAcademic Editor: Martin Goette

Publication date: 2009
Electronic publication date: 2009 Feb 15
Volume: 9
First page: 130
Last page: 132
Received 2008 Sep 25; Revised 2008 Dec 16; Accepted 2009 Jan 14
Copyright: Copyright © 2009 Thian-Sze Wong et al.
Copyright year: 2009
License: This is an open access article distributed under the Creative Commons Attribution License, which permits unrestricted use, distribution, and reproduction in any medium, provided the original work is properly cited.
License URL: https://creativecommons.org/licenses/by/3.0/

Keywords: microRNA, post-transcriptional regulators, squamous cell carcinoma, tongue cancer

==============================
Directions in Science
*TheScientificWorldJOURNAL* (2009) 9, 130–132
ISSN 1537-744X; DOI 10.1100/tsw.2009.12

TheScientificWorldJOURNAL
www.thescientificworld.com

### Mature miR-184 and Squamous Cell Carcinoma of the Tongue

Thian-Sze Wong*, Wai-Kuen Ho, Jimmy Yu-Wai Chan, Raymond Wai-Man Ng, and William Ignace Wei

*Department of Surgery, Faculty of Medicine, The University of Hong Kong*

**E-mail**: thiansze@graduate.hku.hk; wkho@hkucc.hku.hk; jywchan@hotmail.com; ngwmr@hkucc.hku.hk; hrmswwi@hkucc.hku.hk

*Received September 25, 2008; Revised December 16, 2008; Accepted January 14, 2009; Published February 15, 2009*

**KEYWORDS**: microRNA, post-transcriptional regulators, squamous cell carcinoma, tongue cancer

MicroRNAs (miRNAs) are a group of small noncoding RNAs (about 18–24 nucleotides in length) that function as post-transcriptional regulators in eukaryotic cells[1]. This small RNA can bind to the 3'UTR of the target mRNA[2]. Binding of the miRNA can hinder translation of mRNA by promoting degradation or inducing deadenylation[3]. Dysregulation of miRNA expression is thought to play a part in abnormal gene expression in cancer cells[8,12]. miRNAs are transcribed by RNA polymerase II and III[4]. The primary miRNAs are long precursor RNA molecules. Before being transported into the cytoplasm, the precursor molecules are processed by cellular nucleases, such as Drosha, into shorter pre-miRNAs. In the cytoplasm, the pre-miRNAs are processed by RNA III (Dicer) into the functional mature form (single-stranded, ~22 nucleotides)[5]. Mature miRNAs are usually associated with a cellular complex that is similar to the RNA-induced silencing complex that participates in RNA interference[6,7].

We recently evaluated the miRNA expression patterns in squamous cell carcinoma (SCC) of the tongue[8]. Of the 156 examined human mature miRNAs, miR-184 was significantly increased in the tumor cells in comparison with the normal epithelial cells of the tongue. High miR-184 levels were not only detected in the tumor tissues, but also in the plasma of patients with tongue SCC. Decreased plasma levels of miR-184 were observed in patients after surgical removal of the primary tumor. The presentation of miR-184 in our patient samples prompted us to suggest that it is a potential oncogenic miRNA in tongue SCC. We also evaluated the functional role of miR-184 in tongue SCC. We observed that inhibiting miR-184 would promote apoptosis as well as hinder cell proliferation in cultured tongue SCC cells.

The first identified miRNA, lin-4, was discovered in 1993 as a small noncoding RNA that regulates gene expression in *Caenorhabditis elegans*[9]. The second identified miRNA, lethal-7 (let-7), was also found in *C. elegans* in 2000[10]. Later on, similar miRNAs were identified in the human genome. In cancer cells, miRNAs could act as tumor suppressors or oncogenes. Tumor-suppressing miRNAs could inhibit the expression of oncogenic genes being overexpressed in cancers. For example, in patients with chronic lymphocytic leukemia (CLL), suppression of tumor-suppressing miRNAs miR-15A and miR-16-1 results in the up-regulation of the B-cell CLL/lymphoma 2 (BCL2) gene that controls cell proliferation in CLL[11]. In comparison with the tumor-suppressive miRNAs, oncogenic miRNAs could promote tumorigenesis by down-regulating the tumor-suppressor genes. For example, overexpression of the

*Corresponding author.
©2009 with author.
Published by TheScientificWorld; www.thescientificworld.com oncogenic miRNA mir-17-92 cluster could promote cell proliferation by targeting apoptosis-inducing genes[12]. miRNA profiling is now carried out in different human malignancies. Similar to the profiling of gene expression patterns, we see that different cancer types could be distinguished by their differential miRNA expression patterns.

Over the past decades, only a small proportion of miRNAs has been evaluated in human cancer cells. For miR-184, only minimal information is available. Expression of miR-184 in mammalian cells was reported in 2006. High expression was observed in suprabasal cells of the corneal epithelium in the mouse model[13]. Strong expression of miR-184 was observed in the epithelial cells of the cornea in comparison with the germinative layers. Similar to other oncogenic miRNAs, the regulatory mechanism for miR-184 is not yet elucidated. Recently, Nomura et al. established a linkage between miR-184 expression and methylation-imprinting miR-184 in the mouse model[14]. They identified that expression of miR-184 is partly regulated by the methyl CpG-binding protein 2 (MeCP2), a transcription repressor that binds to the methylated CpG sequence. Since methylation imbalance is a common feature of cancers cells, it is interesting to examine the methylation status of the regulatory regions of human miR-184 and examine the correlations with the expression of miR-184.

Our previous understanding of the central dogma of genetics contains only three elements: DNA, RNA, and protein. We typically employ dysregulation or altered function in these three elements to explain the abnormal phenotype of cancer cells. However, we may encounter unexplained situations in which errors or abnormalities in these three elements are not identified. In the future, new insights or answers might be found with the development of epigenetics and the identification of miRNAs. Further studies are warranted to establish the role of miRNAs in cancers and the possible intercorrelation with the central dogma of genetics.

### REFERENCES

1. Cullen, B.R. (2004) Transcription and processing of human microRNA precursors. *Mol. Cell* **16(6),** 861–865.
2. Hutvágner, G. and Zamore, P.D. (2002) RNAi: nature abhors a double-strand. *Curr. Opin. Genet. Dev.***12(2),** 225–232.
3. Hutvágner, G. and Zamore, P.D. (2002) A microRNA in a multiple-turnover RNAi enzyme complex. *Science* **297(5589),** 2056–2060.
4. Lee, Y., Kim, M., Han, J., Yeom, K.H., Lee, S., Baek, S.H., and Kim, V.N. (2004) MicroRNA genes are transcribed by RNA polymerase II. *EMBO J.* **23(20),** 4051–4060.
5. Ketting, R.F., Fischer, S.E., Bernstein, E., Sijen, T., Hannon, G.J., and Plasterk, R.H. (2001) Dicer functions in RNA interference and in synthesis of small RNA involved in developmental timing in C. elegans. *Genes Dev.* **15(20),** 2654–2659.
6. Yi, R., Qin, Y., Macara, I.G., and Cullen, B.R. (2003) Exportin-5 mediates the nuclear export of pre-microRNAs and short hairpin RNAs. *Genes Dev.* **17(24),** 3011–3016.
7. Zeng, Y., Yi, R., and Cullen, B.R. (2003) MicroRNAs and small interfering RNAs can inhibit mRNA expression by similar mechanisms. *Proc. Natl. Acad. Sci. U. S. A.* **100(17),** 9779–9784.
8. Wong, T.S., Liu, X.B., Wong, B.Y., Ng, R.W., Yuen, A.P., and Wei, W.I. (2008) Mature miR-184 as potential oncogenic microRNA of squamous cell carcinoma of tongue. *Clin. Cancer Res.* **14(9),** 2588–2592.
9. Lee, R.C., Feinbaum, R.L., and Ambros, V. (1993) The C. elegans heterochronic gene lin-4 encodes small RNAs with antisense complementarity to lin-14. *Cell* **75,** 843–854.
10. Reinhart, B.J., Slack, F.J., Basson, M., Pasquinelli, A.E., Bettinger, J.C., Rougvie, A.E., Horvitz, H.R., and Ruvkun, G. (2000) The 21-nucleotide let-7 RNA regulates developmental timing in Caenorhabditis elegans. *Nature* **403,** 901–906.
11. Cimmino, A., Calin, G.A., Fabbri, M., Iorio, M.V., Ferracin, M., Shimizu, M., Wojcik, S.E., Aqeilan, R.I., Zupo, S., Dono, M., Rassenti, L., Alder, H., Volinia, S., Liu, C.G., Kipps, T.J., Negrini, M., and Croce, C.M. (2005) miR-15 and miR-16 induce apoptosis by targeting BCL2. *Proc. Natl. Acad. Sci. U. S. A.* **102(39),** 13944–13949.
12. Hayashita, Y., Osada, H., Tatematsu, Y., Yamada, H., Yanagisawa, K., Tomida, S., Yatabe, Y., Kawahara, K., Sekido, Y., and Takahashi, T. (2005) A polycistronic microRNA cluster, miR-17-92, is overexpressed in human lung cancers and enhances cell proliferation. *Cancer Res.***65(21),** 9628–9632.
13. Ryan, D.G., Oliveira-Fernandes, M., and Lavker, R.M. (2006) MicroRNAs of the mammalian eye display distinct and overlapping tissue specificity. *Mol. Vis.* **12,** 1175–1184.
14. Nomura, T., Kimura, M., Horii, T., Morita, S., Soejima, H., Kudo, S., and Hatada, I. (2008) MeCP2-dependent repression of an imprinted miR-184 released by depolarization. *Hum. Mol. Genet.* **17(8),** 1192–1199.

**This article should be cited as follows:**

Wong, T.-S., Ho, W.-K., Chan, J.Y.-W., Ng, R.W.-M., and Wei, W.I. (2009) Mature miR-184 and squamous cell carcinoma of the tongue. *TheScientificWorldJOURNAL* **9**, 130–132. DOI 10.1100/tsw.2009.12.
